# Haplotype-based study of the association of alcohol and acetaldehyde-metabolising genes with alcohol dependence (with or without comorbid anxiety symptoms) in a Cape Mixed Ancestry population

**DOI:** 10.1007/s11011-014-9503-x

**Published:** 2014-02-25

**Authors:** Andrew Crawford, Shareefa Dalvie, Sarah Lewis, Anthony King, Israel Liberzon, George Fein, Karestan Koenen, Rajkumar Ramesar, Dan J. Stein

**Affiliations:** 1 School of Social and Community Medicine, University of Bristol, Oakfield House, Oakfield Grove, Bristol, BS8 2BN UK; 2 MRC Human Genetics Research Unit, Division of Human Genetics, Institute of Infectious Disease and Molecular Medicine, University of Cape Town, Cape Town, South Africa; 3 Department of Psychiatry, University of Michigan, Ann Arbor, USA; 4 VA Ann Arbor Healthcare System, Ann Arbor, MI USA; 5 Neurobehavioral Research Inc, Honolulu, USA; 6 Mailman School of Public Health, Columbia University, New York, USA; 7 Department of Psychiatry and Mental Health, University of Cape Town, Cape Town, South Africa

**Keywords:** Alcohol dependence, Anxiety, Genetics

## Abstract

Alcohol dependence (AD) has a large heritable component. Genetic variation in genes involved in the absorption and elimination of ethanol have been associated with AD. However, some of these polymorphisms are not present in an African population. Previous studies have reported that a type of AD which is characterized by anxious behaviour may be a genetically specific subtype of AD. We investigated whether variation in genes encoding cytochrome P450 2E1 (CYP2E1) or acetaldehyde-metabolising enzymes (ALDH1A1, ALDH2) might alter the risk of AD, with and without symptoms of anxiety, in a Cape population with mixed ancestry. Eighty case control pairs (one with AD, one without AD) were recruited and individually matched for potential confounders. Genotype data were available for 29 single-nucleotide polymorphisms (SNPs) across the three genes. Linkage disequilibrium D′ values were evaluated for all pairwise comparisons. Allele and haplotype frequencies were compared between cases and controls using a *χ* ^2^ test. The ACAG haplotype in block 4 of the ALDH1A1 gene provided evidence of an association with AD (*p* = 0.03) and weak evidence of an association with AD without symptoms of anxiety (*p* = 0.06). When a genetic score was constructed using SNPs showing nominal evidence of association with AD, every extra risk allele increased the odds of AD by 35 % (OR 1.35, 95%CI 1.08, 1.68, *p* = 0.008) and the odds of having AD with anxiety symptoms increased by 53 % (OR 1.53, 95%CI 1.14, 2.05, *p* = 0.004). Although our results are supported by previous studies in other populations, they must be interpreted with caution due to the small sample size and the potential influence of population stratification.

## Introduction

Alcohol dependence (AD) is substantially heritable with studies estimating that heritability is between 50 and 70 % (Heath et al. 1997; Hiroi and Agatsuma 2005; Ystrom et al. 2011; Young-Wolff et al. 2012). Genes associated with AD include those coding for enzymes involved in the absorption and elimination of ethanol such as alcohol dehydrogenase (ADH), aldehyde dehydrogenase (ALDH) and cytochrome P450 2E1 (CYP2E1) (Zakhari 2006; Edenberg 2007). Metabolism of ethanol consists of two rate limiting reactions (Zakhari 2006). Firstly, ethanol is converted to acetaldehyde, which is subsequently metabolized to acetate. The first step is predominantly catalysed by alcohol dehydrogenases (ADH), with minor roles for cytochrome P450 2E1 (CYP2E1) and catalase. In the second step, acetaldehyde is metabolised by aldehyde dehydrogenases (ALDH). Acetaldehyde is considerably more toxic than ethanol, and its accumulation leads to a highly aversive reaction that includes anxiety, facial flushing, nausea, and rapid heartbeat (Eriksson 2001).

Genetic variants that cause a build up of acetaldehyde, either by rapid ethanol metabolism or reduced acetaldehyde metabolism, have been found to be associated with lower risk for AD and heavy drinking (Edenberg 2007). The frequency of these genetic variants varies between ancestral groups (Edenberg 2012). The two polymorphisms that have been most strongly associated with AD in Asian populations, ADH1B Arg47His (rs1229984) and ALDH2 Glu487Lys (rs671), have little/no variation in one African population (Goedde et al. 1992).

AD is a heterogeneous disorder, highly comorbid with internalising disorders (Kessler et al. 1996). Various subtypes of AD have previously been described each with different reasons for developing an addiction, different withdrawal syndromes, different prognoses, and different responses to therapeutic approaches (Lesch et al. 1988). Research has suggested there may be an anxious subtype of AD characterized by high harm avoidance, high reward dependence, and low novelty-seeking behaviour (Cloninger 1987). More recently, reports have suggested this anxious AD may be a genetically specific subtype of AD (Lee et al. 2010). Therefore, if genetic markers could be used to identify this subtype of AD, patient care could be improved by tailoring treatment accordingly.

The median age of onset for AD (23 years of age) is much later than for anxiety disorders (11 years of age) (Kessler et al. 2005). The risk of lifetime dependence to alcohol is far greater for individuals who start drinking at an earlier age (Grant and Dawson 1997). An adolescent cohort of individuals with AD indentifies the most serious cases of AD, and anxiety symptoms would be expected to have been reported by this age.

We investigated whether variation in genes encoding CYP2E1 or acetaldehyde-metabolising enzymes (ALDH1A1, ALDH2) might alter the risk of AD in an adolescent Cape population with mixed ancestry by performing systematic haplotype association analyses to maximize the chances of capturing functional variation. We also investigated the association between a genotype risk score and AD. Investigating genetic associations in different population groups is important in order to replicate and validate previous findings, or where results do not correlate it may indicate heterogeneity. Additionally, we investigated whether AD with or without comorbid symptoms of anxiety may be a genetically specific subtype of AD.

## Methods

### Participants

Details of the participants have been reported previously (Ferrett et al. 2011). In brief, 80 case control pairs (one with AD, one without AD) from within the Cape Flats region (Cape Town, South Africa) were individually matched for age (within 1 year), gender (each group consisted of 47 females and 33 males), education level, language and socioeconomic status (SES). The average participant was aged 14.8 years (sd 0.76) and had completed 7.6 years (sd 0.82) of education. The sample reflected the sociodemographic profile of the Cape Flats population (100 % Coloured; Language, 69 % Afrikaans, 31 % English; 86 % in households with formal housing; and 85 % earning a gross annual income of less than ZAR 100 000). Exclusion criteria included, but were not limited to: mental retardation; lifetime DSM-IV Axis I diagnoses other than AD (including the following disorders: depressive, anxiety, psychotic, post-traumatic stress, eating, tic, attention-deficit/hyperactivity, oppositional defiant, and conduct); less than 6 years of formal education; and lack of proficiency in English or Afrikaans. Volunteers were screened for eligibility after written informed assent/consent was obtained from volunteers and parents or guardians.

The study protocol and procedures complied with and were conducted in strict adherence to the guidelines contained in the Declaration of Helsinki (2008). Full written approval to conduct the study was obtained from the Western Cape Education Department and the Research Ethics Committee of the Stellenbosch University Faculty of Health Sciences.

### Measures

#### Alcohol use

A revised version of the Timeline Followback (TLFB) procedure (Sobell and Sobell 1992), a semi-structured, clinician-administered assessment of alcohol use and drinking patterns, was used in collaboration with the Kiddie Schedule for Affective Disorders and Schizophrenia Present and Lifetime Versions (K-SADS-PL) (Kaufman et al. 1997) to elicit alcohol-use data. A standard drink was defined as one beer or wine cooler, one glass of wine, or one 1.5-oz shot of liquor (alone or in a mixed drink). AD was defined by a lifetime dosage in excess of 100 units plus a DSM-IV diagnosis of alcohol abuse or dependence. The control group were non-drinkers (who had never consumed alcohol) and light drinkers (lifetime dosage not exceeding 76 units of alcohol), with no history of AD.

#### Psychopathology

Total symptom counts from the K-SADS-PL were recorded for generalised anxiety disorder. As previously mentioned, individuals with a diagnosis of anxiety disorders were excluded from the study. However, individuals reporting low levels of anxiety symptoms, not severe enough for a diagnosis of anxiety disorders, were included in the study. A binary variable was generated for the presence or absence of these anxiety symptoms in individuals with AD (anxious-AD). Of the 80 individuals with AD, there were 59 individuals without any anxiety symptoms and 21 with anxiety symptoms.

#### Genotyping

There were genotype data on a total of 29 single-nucleotide polymorphisms (SNPs) (16 SNPs in ALDH1A1, 7 SNPs in ALDH2, 6 SNPs in CYP2E1). Genotyping was carried out using a custom Illumina Infinium iSelect custom 6000 bead chip.

#### Genotype risk score

We calculated a genotype risk score using all SNPs moderately associated with outcome (chi-square value greater than 1). The score was the unweighted sum of the number of risk alleles (0, 1 or 2) at each of these SNP loci. Separate genotype risk scores were created for the outcomes of AD and anxious-AD.

### Statistical analysis

The genotype distributions for each SNP in the control group (without AD) were used to calculate deviation from Hardy–Weinberg equilibrium (HWE) using a *χ* ^2^ test, and those SNP’s which did show evidence of deviation were excluded from further analysis. Linkage disequilibrium (LD) D′ values were evaluated for all marker pairs. Customised haplotype blocks were defined in Haploview version 4.2 (Barrett et al. 2005). Allele and haplotype frequencies were compared between cases and controls using a *χ* ^2^ test. Logistic regression models were used to investigate the association between genotype risk score and the outcomes of AD, or AD with anxiety symptoms. A Bonferroni correction was applied to address the issues associated with multiple testing (Bland and Altman 1995). A power calculation was performed using the Quanto software (Version 1.2.4) (Gauderman and Morrison 2006). Statistical analyses were performed using Haploview version 4.2 (Barrett et al. 2005) and Stata version 12.1 (StataCorp 2011).

## Results

There was evidence that one SNP (rs348457) deviated from HWE (*p* < 0.001) and there was no genetic variation in another SNP (rs671). Both these SNPs were excluded from our analyses. This left a total of 27 SNPs in the analysis (15 for ALDH1A1, 6 for ALDH2, 6 for CYP2E1). The allele frequencies for the 27 SNPs are presented in Table 1. To correct for the multiple testing of 27 SNPs in two disease models, a threshold level of significance was calculated as *p* < 0.0009. This is a conservative estimate due to the LD between SNPs. Given the number of tests, there was no evidence of any associations other than one would expect by chance.

**Table 1:** Allele frequencies and associations with alcohol dependence or alcohol dependence with anxiety symptoms

Gene	SNP	Allele	AD			AD with anxiety symptoms		
			Case, control frequencies	Chi square	*P* value	Case, control frequencies	Chi square	*P* value
ALDH1A1	rs8187998	A	1.000, 0.994	1.00	0.32	1.000, 1.000	−	−
	rs1888202	C	0.625, 0.619	0.01	0.91	0.548, 0.653	1.46	0.23
	rs63319	A	0.538, 0.531	0.01	0.91	0.429, 0.576	2.72	0.10
	rs8187974	A	0.006, 0.000	1.00	0.32	0.000, 0.008	0.36	0.55
	rs2773806	G	0.256, 0.206	1.13	0.29	0.262, 0.254	0.01	0.92
	rs1424482	G	0.506, 0.494	0.05	0.82	0.500, 0.508	0.01	0.92
	rs8187876	G	0.850, 0.844	0.02	0.88	0.857, 0.847	0.02	0.88
	rs11143429	A	0.644, 0.631	0.05	0.82	0.643, 0.644	0.00	0.99
	rs6560311	C	0.719, 0.669	0.94	0.33	0.738, 0.712	0.11	0.75
	rs2249978	G	0.519, 0.506	0.05	0.82	0.500, 0.525	0.08	0.78
	rs1418187	G	0.612, 0.588	0.21	0.65	0.571, 0.627	0.41	0.52
	rs4745209	G	0.338, 0.325	0.06	0.81	0.381, 0.322	0.48	0.49
	rs7860980	C	0.956, 0.938	0.56	0.45	0.952, 0.958	0.02	0.89
	rs4406477	A	0.569, 0.556	0.05	0.82	0.548, 0.576	0.10	0.75
	rs11143443	G	0.138, 0.100	1.08	0.30	0.071, 0.161	2.10	0.15
ALDH2	rs2238151	G	0.219, 0.219	0.00	0.99	0.857, 0.754	1.92	0.17
	rs2238152	A	0.162, 0.138	0.39	0.53	0.167, 0.161	0.01	0.93
	rs4648328	A	0.162, 0.131	0.62	0.43	0.167, 0.161	0.01	0.93
	rs7311852	C	0.031, 0.006	2.72	0.10	0.024, 0.034	0.10	0.75
	rs4646778	A	0.162, 0.138	0.39	0.53	0.167, 0.161	0.01	0.93
	rs7296651	C	0.594, 0.512	2.14	0.14	0.667, 0.568	1.26	0.26
CYP2E1	rs3813865	G	0.825, 0.819	0.02	0.88	0.857, 0.814	0.41	0.52
	rs3813867	G	0.056, 0.050	0.06	0.80	0.024, 0.068	1.13	0.29
	rs915906	A	0.544, 0.488	1.01	0.31	0.619, 0.517	1.30	0.25
	rs6413419	G	0.931, 0.862	4.09	0.04	0.929, 0.932	0.01	0.94
	rs743535	G	0.831, 0.819	0.09	0.77	0.857, 0.822	0.27	0.60
	rs2515642	A	0.569, 0.569	0.00	0.99	0.524, 0.398	1.99	0.16

*AD* alcohol dependence. A total of 160 individuals were included in the AD analysis (80 with AD, 80 without AD). A total of 80 AD individuals were included in the AD with anxiety analysis (21 with anxiety symptoms, 59 without anxiety symptoms)

### Linkage disequilibrium

**Fig. 1:**
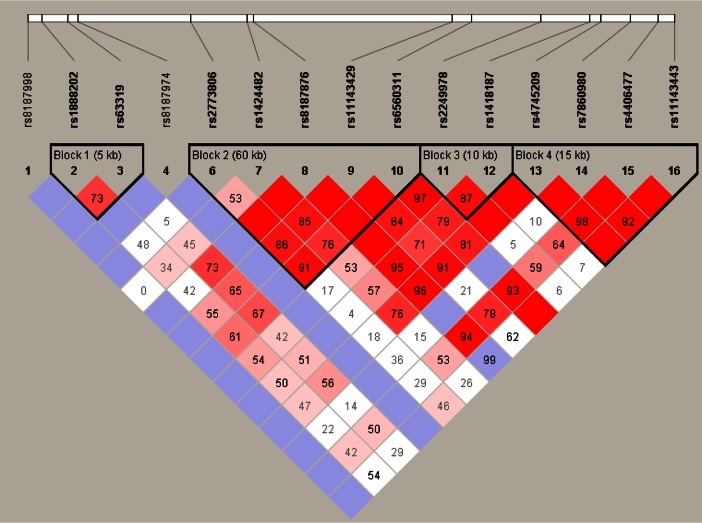
Haplotype block structure for the ALDH1A1 gene on chromosome 9. Haplotype blocks are outlined. Figures represent D′

**Fig. 2:**
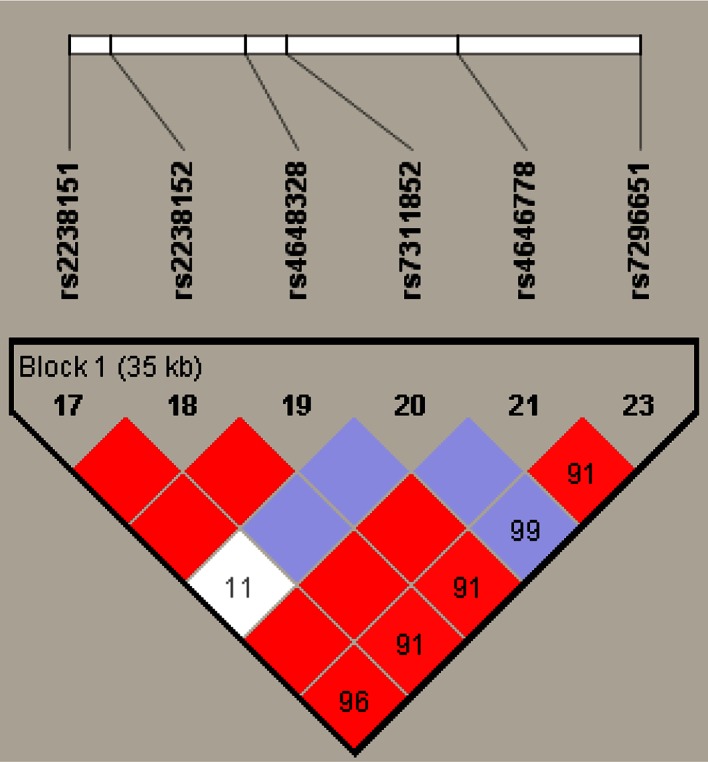
Haplotype block structure for the ALDH2 gene on chromosome 12. Haplotype blocks are outlined. Figures represent D′

**Fig. 3:**
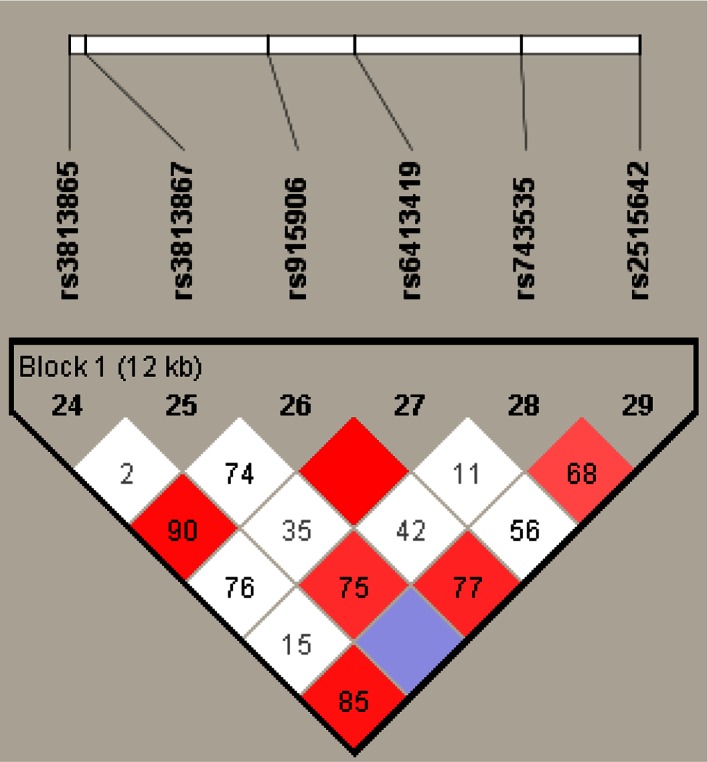
Haplotype block structure for the CYP2E1 gene on chromosome 10. Haplotype blocks are outlined. Figures represent D′

The extent of LD between the SNPs was determined for ALDH1A1 (Fig. 1), ALDH2 (Fig. 2) and CYP2E1 (Fig. 3).

### Alcohol dependence analysis

A total of 160 individuals were included in the analysis (80 with AD, 80 without AD). There was some evidence of an association between AD and rs6413419 in the CYP2E1 gene (*p* = 0.04) (Table 1). In the haplotype analysis there was some evidence of an association with the ACAG haplotype in block 4 of the ALDH1A1 gene (*p* = 0.03) (Table 2).

**Table 2:** ALDH1A1, ALDH2 and CYP2E1 haplotype frequencies and associations with alcohol dependence or alcohol dependence with anxiety symptoms

Gene	Block	Haplotype	Freq.	AD			AD with anxiety symptoms		
				Case control frequencies	Chi square	*P* value	Case control frequencies	Chi square	*P* value
ALDH1A1	Block1	CA	0.48	0.491, 0.469	0.16	0.69	0.395, 0.525	2.12	0.15
		GC	0.32	0.329, 0.319	0.04	0.85	0.418, 0.297	2.08	0.15
		CC	0.14	0.134, 0.150	0.17	0.68	0.153, 0.127	0.18	0.67
		GA	0.05	0.046, 0.062	0.40	0.53	0.034, 0.051	0.20	0.66
	Block 2	AAGGC	0.33	0.332, 0.332	0.00	0.99	0.355, 0.324	0.13	0.72
		GGGAC	0.16	0.191, 0.137	1.72	0.19	0.198, 0.189	0.02	0.89
		AGAAA	0.15	0.150, 0.156	0.02	0.88	0.143, 0.153	0.02	0.88
		AGGAA	0.10	0.089, 0.108	0.33	0.56	0.075, 0.094	0.15	0.70
		AAGAC	0.08	0.089, 0.066	0.56	0.46	0.042, 0.105	1.55	0.21
	Block 3	GG	0.49	0.503, 0.472	0.31	0.58	0.496, 0.505	0.01	0.91
		AA	0.38	0.372, 0.378	0.02	0.90	0.424, 0.353	0.68	0.41
		AG	0.11	0.110, 0.116	0.03	0.87	0.076, 0.122	0.67	0.41
	Block 4	ACAA	0.44	0.425, 0.456	0.32	0.57	0.476, 0.406	0.61	0.44
		GCGA	0.32	0.324, 0.324	0.00	0.99	0.380, 0.305	0.81	0.37
		ACGA	0.11	0.100, 0.119	0.29	0.59	0.072, 0.111	0.51	0.48
		ACAG	0.07	0.100, 0.038	4.88	0.03	0.024, 0.127	3.68	0.06
		AAAG	0.05	0.037, 0.062	1.05	0.30	0.048, 0.034	0.17	0.68
ALDH2	Block 1	GCGGCC	0.39	0.402, 0.384	0.11	0.74	0.479, 0.375	1.42	0.23
		GCGGCG	0.23	0.193, 0.260	2.03	0.15	0.189, 0.195	0.01	0.93
		ACGGCG	0.21	0.211, 0.212	0.00	0.98	0.141, 0.236	1.66	0.20
		GAAGAC	0.14	0.153, 0.122	0.65	0.42	0.162, 0.149	0.04	0.84
CYP2E1	Block 1	GCAGGA	0.38	0.390, 0.371	0.12	0.73	0.472, 0.360	1.63	0.20
		GCGGGG	0.15	0.157, 0.150	0.03	0.87	0.130, 0.167	0.33	0.57
		CCGGGG	0.11	0.124, 0.104	0.32	0.57	0.082, 0.139	0.91	0.34
		GCGAGG	0.07	0.050, 0.094	2.30	0.13	0.048, 0.051	0.01	0.93
		GCAGGG	0.07	0.067, 0.070	0.02	0.90	0.096, 0.056	0.79	0.37

*AD* alcohol dependence. The table lists haplotypes that have a frequency greater than 5 %. A total of 160 individuals were included in the AD analysis (80 with AD, 80 without AD). A total of 80 AD individuals were included in the AD with anxiety analysis (21 with anxiety symptoms, 59 without anxiety symptoms)

### Alcohol dependence with anxiety symptoms analysis

A total of 80 individuals with AD were included in the analysis (21 with anxiety symptoms, 59 without anxiety symptoms). There was weak evidence of an association with anxious-AD and rs63319 of the ALDH1A1 gene (*p* = 0.10) (Table 1). In the haplotype analysis there was weak evidence of an association with the ACAG haplotype in block 4 of the ALDH1A1 gene (*p* = 0.06) (Table 2).

### Genotype risk score

There were 8 SNPs (4 SNPs in ALDH1A1, 2 SNPs in ALDH2, 2 SNPs in CYP2E1) associated with AD that were included in this genotype risk score. This score ranged from 5 to 13. For every increase in genotype risk score (ie. for every extra risk allele) the odds of AD increased by 35 % (OR 1.35, 95%CI 1.08, 1.68, *p* = 0.008).

There were 8 SNPs (3 SNPs in ALDH1A1, 2 SNPs in ALDH2, 3 SNPs in CYP2E1) associated with anxious-AD that were included in this genotype risk score. This score ranged from 5 to 16. For every increase in genotype risk score (ie. for every extra risk allele) the odds of having AD with anxiety symptoms (rather than AD without anxiety symptoms) increased by 53 % (OR 1.53, 95%CI 1.14, 2.05, *p* = 0.004). The effect of both genotype risk scores appeared to be linear, although interpretation is limited due to a small number of individuals at the extremes.

### Power calculation

We performed a post hoc statistical power and sample size analysis. Statistical power is defined as the probability of rejecting the null hypothesis while the alternative hypothesis is true. The results vary for each SNP investigated but assuming an allele frequency of 0.9 (rs6413419), a population risk of 0.1, an additive genetic model, an odds ratio of 3 (aa v. AA) and significance set at 5 %, we had 27 % power. Using the same assumptions but setting the power to 80 % we would need a sample size approximately five times the size of the current study (or ten times the size for the anxious-AD analysis).

## Discussion

### Main findings and comparisons with the literature

Although AD is prevalent in South Africa (Williams et al. 2008; Peltzer et al. 2011) there has been a paucity of previous research investigating genetic variants associated with this phenotype in a South African population. The ACAG haplotype in block 4 of the ALDH1A1 gene had a frequency of 6.9 % in our cohort and provided some evidence of an association with AD. This haplotype was more frequent in individuals with AD than controls (10 % v. 3.8 %, *p* = 0.03) and more frequent in individuals with AD but without anxiety symptoms than individuals with AD and anxiety symptoms (12.7 % v. 2.4 %, *p* = 0.06). Therefore, it is possible that this haplotype may identify non-anxious AD as a genetically specific subtype of AD. There were also encouraging findings with regard to the genotype risk score.

The association between AD and the ACAG haplotype was driven by rs11143443. This SNP is located upstream of the 5′ promoter region of the ALDH1A1 gene and has previously been associated with AD in an African American population (Liu et al. 2011). Genetic variation in the promoter region has been reported to affect ALDH1A1 gene expression (Spence et al. 2003), although to our knowledge no such gene expression data exist for this particular SNP. Liu et al. (Liu et al. 2011) also reported evidence of an association between AD and a haplotype in ALDH2, driven by rs7311852. The same SNP provided weak evidence of an association with AD in our data. However, the authors report that the positive association was influenced by population stratification.

The Cape Mixed Ancestry group, which has been shown to have the greatest level of intercontinental admixture compared to any other international population group, consists of individuals of Khoesan, Bantu-speaking African, European and Asian ethnicity (Tishkoff et al. 2009; de Wit et al. 2010). A principal components analysis, using genetic markers across the genome, would be required to determine whether this cohort consists of a single genetic population, and thus whether our associations are influenced by population stratification. Comparing the allele frequencies of individuals in this study with populations in the HapMap project (Gibbs et al. 2003) showed the frequencies to be intermediate between the Utah residents with ancestry from northern and western Europe (CEU) and the Yoruba in Ibadan, Nigeria (YRI) (data not shown).

### Strengths, limitations and future directions

The main limitation of this study is the small sample size which is further compounded in our pre-specified subgroup analysis of AD with or without anxiety symptoms. Therefore, our findings must be interpreted with caution and should be considered preliminary. This is especially true given that there was no evidence of any association other than that which one would expect by chance. However, we limited our analyses to focus on three biologically relevant genes and have been clear on the tests that have been performed which is generally more important when dealing with multiple comparisons (Perneger 1998).

This adolescent cohort contained extremely well defined cases of AD as individuals with a diagnosis of comorbid generalised anxiety disorder were excluded from the study. Therefore, our power to identify a genetically specific subtype of non-anxious AD will be greater than other cohorts of a similar size. Our power calculation clearly indicates that larger studies are required in this area. A potential limitation of this selective cohort is that the results may not be generalisable to the general population where AD tends to be highly comorbid with other disorders. Additionally, as this is an adolescent cohort, and the median age of onset for AD is 23 years of age, it is possible that individuals included as controls in this study may go on to develop AD. However, AD is inversely associated with age of first drink and so individuals with AD in this cohort are likely to have a more severe phenotype than a control individual that goes onto develop AD at a later stage (Grant and Dawson 1997).

For some SNPs the evidence of an association was stronger in the AD analysis than in the anxious-AD analysis. This may be because this is a marker associated with AD in general rather than with a specific subtype of AD. Alternatively, this may also be explained by the reduction in sample size between the two analyses. This can be seen for the SNP rs7296651 where the effect size in the AD analysis is less extreme, but the strength of evidence for the association is stronger than in the anxious-AD analysis (AD analysis: OR 0.71, 95%CI 0.45 to 1.12, *p* = 0.14, compared with anxious-AD analysis: OR 0.62, 95%CI 0.28 to 1.37, *p* = 0.24). The same set of individuals were used in the discovery and testing of the genetic risk scores therefore, although we observed relatively large effect sizes future work could look to validate these findings in an independent sample.

This study increases the body of evidence investigating genetic variants and AD in a genetically admixed population. It is important to investigate this type of population in order to replicate previous findings, as well as attempting to identify genetic variants for complex disorders (de Wit et al. 2010). Meta-analyses of genetic studies will be needed to identify genetically-specific subtypes of AD, potentially providing insights into the biological mechanism of these disorders.
